# High Level of the Fibrin Degradation Products at Admission Predicts Parenchymal Hematoma and Unfavorable Outcome of Ischemic Stroke After Intravenous Thrombolysis

**DOI:** 10.3389/fneur.2021.797394

**Published:** 2022-01-18

**Authors:** Chang Liu, Yun Zhang, Lingchuan Niu, Jiani Li

**Affiliations:** ^1^Department of Neurology, The Second Affiliated Hospital of Chongqing Medical University, Chongqing, China; ^2^Department of Rehabilitation Medicine, The Second Affiliated Hospital of Chongqing Medical University, Chongqing, China

**Keywords:** acute ischemic stroke, fibrin degradation products, parenchymal hematomas, outcome, intravenous thrombolysis

## Abstract

**Background and Purpose:**

We aim to investigate whether the higher admission fibrin degradation products (FDPs) levels are associated with parenchymal hematomas (PHs) and unfavorable outcome after intravenous thrombolysis (IVT).

**Methods:**

Consecutive patients with acute ischemic stroke treated with IVT were studied. The FDP level was obtained on admission. PH was evaluated 24 h after treatment. The unfavorable outcome was defined as a 90-day modified Rankin Scale >2. The multivariable linear stepwise regression was used to assess independent factors associated with the log-transformed FDP (lgFDP). The receiver operating characteristics (ROCs) curve analysis was used to determine the predictive value of the FDP level for PH and unfavorable outcome. The logistic regression was used to identify independent predictors for PH and unfavorable outcome. The mediation analyses were performed to investigate associations among the FDP level, PH, and outcome.

**Results:**

A total of 181 patients were included in the final analyses [median age, 73 (63–79) years; 102 (56.4%) males; and the median baseline National Institutes of Health Stroke Scale (NIHSS) score, 8 (5–15)]. The lgFDP was independently associated with age (*B* = 0.011, 95% CI 0.006–0.015, *p* < 0.001) and the baseline NIHSS score (*B* = 0.016, 95% CI 0.008–0.025, *p* < 0.001). The FDP was positively associated with PH [odds ratio (OR) 1.034, 95% CI 1.000–1.069; *p* = 0.047]. According to the ROC analysis, the best discriminating factor for unfavorable outcome was the FDP ≥3.085 μg/ml. The FDP ≥3.085 μg/ml was an independent predictor of unfavorable outcome (OR 7.086, 95% CI 2.818–17.822; *p* < 0.001). Mediation analysis revealed that the association of the FDP ≥3.085 μg/ml with unfavorable outcome was not mediated by PH (*p* = 0.161).

**Conclusion:**

The admission FDP levels can predict PH and unfavorable outcome in patients with acute ischemic stroke after IVT. PH does not mediate the effect of the FDP level on the outcome.

## Introduction

Stroke is the second leading cause of death worldwide and the leading cause of death in China (1). Intravenous thrombolysis (IVT) with recombinant tissue-type plasminogen activator (rt-PA) within 4.5 h has been a standard treatment for acute ischemic stroke (AIS) nowadays (2). Patients with AIS due to large vessel occlusion can also benefit from timely endovascular thrombectomy (2, 3). Over the past few years, the therapeutic time window of IVT and endovascular thrombectomy had been extended in selected patients (4), so that more patients can benefit from reperfusion therapy and have the opportunity to improve functional outcome. However, one of the main complications of IVT and endovascular thrombectomy is hemorrhagic transformation (HT) (5), especially parenchymal hematomas (PHs), which may be life-threatening. Therefore, it is crucial to detect risk factors of PH and unfavorable outcome after IVT.

The fibrin degradation products (FDPs), a marker of fibrinolytic activity, are fragments released following plasmin-mediated degradation of fibrinogen and fibrin (6). The elevated FDP indicates fibrin/fibrinogen degradation, which is sensitive to intravascular thrombus. A previous study found that the FDP levels and D-dimer levels increased significantly after stroke, thus the authors thought endogenous fibrinolysis was activated after stroke (7). Furthermore, the FDP also plays a role in atherosclerosis, since it can stimulate collagen synthesis, attract leukocytes, and alter endothelial permeability (8). Previous studies had demonstrated that increased FDP levels 2 h post-IVT were associated with PH (9, 10). A recent study found that the admission FDP levels were negatively associated with early thrombolytic effect (11). Nevertheless, few studies focused on the relationships among the admission FDP level, PH, and the 90-day outcome after IVT in patients with AIS. Since almost every patient had coagulation testing, including the FDP, before IVT, it could be helpful, if the admission FDP could predict stroke prognosis. We hypothesized that patients with higher levels of the FDP might be more likely to experience PH and have unfavorable outcome after IVT. Furthermore, we sought to investigate whether PH mediated the relationship between the admission FDP level and the unfavorable outcome.

## Materials and Methods

### Study Subjects and Clinical Data

This was a single-center retrospective study. We reviewed our prospectively collected cohort for consecutive patients with AIS who received IVT with or without endovascular thrombectomy from January 2019 to March 2021. We then enrolled patients who: (1) age > 18 years; (2) received rt-PA within 4.5 h of symptom onset; (3) had the plasma FDP level collected before IVT; and (4) had CT/MRI performed 24 h after IVT. Exclusion criteria were as follows: (1) 90-day modified Rankin Scale (mRS) were unavailable and (2) the baseline mRS > 2. Intravenous alteplase was administered according to the international guidelines (0.9 mg/kg, maximum dose 90 mg, and 10% in a bolus in 1 min with the remaining dose in a 60-min infusion) (2).

Demographic characteristics and baseline clinical information including age; sex; the admission National Institutes of Health Stroke Scale (NIHSS); risk factors of stroke (smoking history, hypertension, diabetes, hyperlipidemia, and atrial fibrillation); and coagulative function (FDP, international normalized ratio, prothrombin time, and activated partial thromboplastin time) were extracted from the electronic records. PH and symptomatic intracranial hemorrhage (SICH) were evaluated 24 h after treatment according to the European Cooperative Acute Stroke Study (ECASS) II criteria. Specifically, PH1 was defined as blood clots in <30% of the infarcted area with some slight space-occupying effect and PH2 was defined as blood clots in more than 30% of the infarcted area with a substantial space-occupying effect (12). SICH was defined as the clinical deterioration (≥4 points in the NIHSS score) due to the hemorrhage (12). HT was assessed by 2 neurologists (CL and YZ, both with more than 2 years of neuroimaging review experience), who were blinded to the clinical data of subjects. Clinical outcomes were evaluated by the mRS at 90-day post-stroke, which were assessed by telephone or face-to-face. The favorable and unfavorable outcomes were defined as mRS ≤ 2 and >2, respectively.

### Statistical Analysis

Median (25 to 75th percentile) for non-normal distributed continuous variables and frequency (percentage) for categorical variables were used. The Spearman correlation analysis was used to assess relationships between the log-transformed FDP (lgFDP) and characteristics of patients. The multivariable linear stepwise regression was used to assess independent factors associated with the lgFDP. The receiver operating characteristics (ROCs) curve analyses were used to determine the predictive value of FDP for PH and unfavorable outcome. The chi-squared test or the Mann–Whitney *U* test was used to compare the variables between the two groups. The logistic regression analyses were performed to assess independent predictors of PH and unfavorable outcome. Variables that had a univariate *p*-value < 0.05 were analyzed in the logistic regression model. Mediation analysis was performed to investigate whether PH (as the mediator) was the driving factor for any relationship between the FDP level (independent variable) and unfavorable outcome (dependent variable) using the Karlson–Holm–Breen (KHB) method (13). A significance level of 0.05 was used to assess the statistical difference. A user-written *khb* command implemented the KHB method in the Stata/SE software version 15.0 (StataCorp LLC, College Station, Texas, USA) (14). All other statistical analyses were performed using the SPSS software version 20.0 (IBM Incorporation, Armonk, New York, USA).

## Results

A total of 181 patients were included in this study. Of these, 146 patients underwent IVT alone and 35 patients had IVT combined with endovascular thrombectomy. The median age was 73 (63–79) years and 102 (56.4%) were males. The median baseline NIHSS score was 8 (5–15). The median FDP was 2.5 (1.5–3.6) μg/ml. In total, 43 (23.8%) patients had HT, among them 27 (14.9%) patients had PH, and 5 (2.8%) patients had SICH. There were 71 (39.2%) patients who had an unfavorable outcome at 90-day post-stroke.

### Relationship Between the FDP and Characteristics of Patients

The admission lgFDP was positively correlated with age (ρ = 0.509, *p* < 0.001), history of atrial fibrillation (ρ = 0.341, *p* < 0.001), and the baseline NIHSS score (ρ = 0.346, *p* < 0.001). Male sex (ρ = −0.266, *p* < 0.001) and smoking (ρ = −0.246, *p* = 0.001) had negative correlations with the lgFDP. However, history of hypertension (ρ = −0.111, *p* = 0.137), diabetes (ρ = 0.040, *p* = 0.596), and hyperlipemia (ρ = −0.107, *p* = 0.152) were not significantly correlated with the lgFDP. In multiple linear regression, the lgFDP was independently associated with age (*B* = 0.011, 95% CI 0.006–0.015, *p* < 0.001) and the baseline NIHSS score (*B* = 0.016, 95% CI 0.008–0.025, *p* < 0.001).

### Association of the FDP and PH

As shown in Table 1, patients who underwent endovascular thrombectomy, with hypertension, the greater baseline NIHSS score, and the higher FDP were more likely to have PH (*p* < 0.05). The ROC analysis revealed acceptable predictive value of the FDP (area under the curve = 0.666, 95% CI 0.550–0.781, *p* = 0.006). The cutoff point of the FDP for PH was 3.140 μg/ml and this yielded a sensitivity of 63.0% and a specificity of 68.8%. In the logistic regression, the FDP [odds ratio (OR) 1.034, 95% CI 1.000–1.069; *p* = 0.047] and endovascular thrombectomy (OR 4.553, 95% CI 1.711–12.115; *p* = 0.002) were independently associated with PH (Supplementary Table 1).

**Table 1:** Univariate comparison between the parenchymal hematomas (PHs) group and the non-PH group.

	**Non-PH (*n* = 154)**	**PH (*n* = 27)**	***P*-value**
Age, y	72.0 (63.0–79.0)	75.0 (62.5–81.5)	0.482
Male, *n* (%)	90 (58.4)	12 (44.4)	0.176
Hypertension, *n* (%)	112 (72.7)	13 (48.1)	0.011
Diabetes, *n* (%)	45 (29.2)	9 (33.3)	0.667
Hyperlipemia, *n* (%)	102 (66.2)	15 (55.6)	0.284
Atrial fibrillation, *n* (%)	59 (38.3)	14 (51.9)	0.186
Smoking, *n* (%)	65 (42.2)	7 (25.9)	0.111
Baseline NIHSS score	8.0 (4.0–13.0)	14.5 (8.0–19.0)	0.001
FDP, μg/ml	2.3 (1.5–3.4)	3.4 (2.0–6.0)	0.006
International normalized ratio	0.98 (0.93–1.05)	1.01 (0.98–1.09)	0.092
Prothrombin time, s	13.0 (12.5–13.7)	13.2 (12.8–14.2)	0.096
Activated partial thromboplastin time, s	34.7 (32.3–37.0)	34.7 (31.4–37.1)	0.612
Endovascular thrombectomy, *n* (%)	22 (14.3)	13 (48.1)	<0.001

*NIHSS, National Institutes of Health Stroke Scale; FDPs, fibrin degradation products*.

### Association of the FDP and Unfavorable Outcome

Patients in the unfavorable outcome group were older and had the greater baseline NIHSS score, the higher FDP, higher international normalized ratio, and longer prothrombin time than patients in the favorable outcome group (*p* < 0.05; Table 2). Moreover, more females and patients with hypertension and atrial fibrillation were in the unfavorable outcome group than the favorable outcome group (*p* < 0.05; Table 2). The ROC analysis revealed an optimal FDP threshold for the unfavorable outcome of 3.085 μg/ml, with the area under the curve of 0.749 (95% CI 0.675–0.823, *p* < 0.001), which yielded a sensitivity of 64.8% and a specificity of 79.1%. We found no significant association between the FDP, as a continuous variable, and unfavorable outcome after adjusting for confounding factors (OR 1.026, 95% CI 0.968–1.087; *p* = 0.391; Supplementary Table 2). However, the FDP > 3.085 μg/ml was an independent predictor of unfavorable outcome (OR 7.086, 95% CI 2.818–17.822; *p* < 0.001; Supplementary Table 2). The baseline NIHSS score (OR 1.185, 95% CI 1.091–1.288; *p* < 0.001) and PH (OR 3.639, 95% CI 1.065–12.441; *p* = 0.039) were associated with unfavorable outcome independently as well (Supplementary Table 2).

**Table 2:** Univariate comparison between the favorable outcome group and the unfavorable outcome group.

	**Favorable outcome (*n* = 110)**	**Unfavorable outcome (*n* = 71)**	***P*-value**
Age, y	71.0 (61.0–78.0)	76.0 (66.5–82.0)	0.004
Male, *n* (%)	71 (64.5)	31 (43.7)	0.006
Hypertension, *n* (%)	83 (75.5)	42 (59.2)	0.021
Diabetes, *n* (%)	33 (30.0)	21 (29.6)	0.952
Hyperlipemia, *n* (%)	71 (64.5)	46 (64.8)	0.973
Atrial fibrillation, *n* (%)	36 (32.7)	37 (52.1)	0.009
Smoking, *n* (%)	48 (43.6)	24 (33.8)	0.187
Baseline NIHSS score	6.0 (4.0–9.0)	14.0 (9.0–18.0)	<0.001
FDP, μg/ml	2.0 (1.3–2.9)	3.4 (2.5–5.4)	<0.001
International normalized ratio	0.98 (0.92–1.03)	0.99 (0.96–1.09)	0.011
Prothrombin time, s	13.0 (12.5–13.6)	13.2 (12.8–14.2)	0.018
Activated partial thromboplastin time, s	34.7 (33.0–36.8)	34.2 (31.8–38.9)	0.059
Endovascular thrombectomy, *n* (%)	13 (11.8)	22 (31.0)	0.001
Parenchymal hematoma, *n* (%)	6 (5.5)	21 (29.6)	<0.001

*NIHSS, National Institutes of Health Stroke Scale; FDPs, fibrin degradation products*.

### Mediation Analysis of the FDP in Unfavorable Outcome by PH

In the mediation analysis for the unfavorable outcome, the FDP > 3.085 μg/ml was defined as the key independent variable and PH as mediator. The baseline NIHSS score was included as the control variable. The direct effect was the effect of the FDP > 3.085 μg/ml on the unfavorable outcome. The indirect effect was the part of the relationship between the FDP and unfavorable outcome that was due to PH. Table 3 shows the results of the decomposition. The total effect of the FDP > 3.085 μg/ml on unfavorable outcome, 1.75, could be decomposed into a direct effect, 1.60 (*p* < 0.001) and indirect effect, 0.15 (*p* = 0.161). Thus, the mediating effect was not statistically significant.

**Table 3:** Mediation analysis of the association of the fibrin degradation product (FDP) >3.085 μg/ml with the unfavorable outcome with parenchymal hematoma as a mediator.

	**B/(SE)**	**95%CI**	*P-*value^*^
Total effect	1.75 (0.39)	0.98–2.51	<0.001
Direct effect	1.60 (0.39)	0.84–2.36	<0.001
Indirect effect	0.15 (0.11)	−0.06 to 0.35	0.161

* *Adjusted for the baseline National Institutes of Health Stroke Scale (NIHSS) score*.

## Discussion

This study found a high level of the FDP associated with PH and unfavorable outcome 90-day after stroke in patients who received IVT. PH did not mediate the relationship between the FDP and the unfavorable outcome. This study indicates that patients with the high FDP levels should be monitored more carefully.

The imbalance of the coagulation/fibrinolysis system is crucial in thrombus formation and progression. The increase of the FDP may represent primary or secondary fibrinolysis in conditions such as hypoxia, infections, and thromboembolic states. The FDP can promote the proliferation of endothelial cells, smooth muscle cells, fibroblast, and cholesterol deposition, which was associated with the development and progression of atherosclerosis (6). Thus, the FDP may play a role in the progression of ischemic stroke.

In this study, older patients and patients with the higher baseline NIHSS score had the higher admission FDP. This suggested that the FDP might associate with stroke severity. The FDP was widely studied as an indicator of disease severity and for differential diagnosis. The FDP was associated with the severity of coronavirus disease 2019 (COVID-19) and trauma (15, 16). In patients with COVID-19, those with the higher FDP had higher risks of stroke (17). Kono et al. found that patients with cancer-associated AIS had the higher FDP than patients with conventional AIS (18). The FDP could also be used as a marker for differential diagnosis between AIS and acute aortic dissection (19, 20).

We found the rates of PH and SICH were 14.9 and 2.8%, respectively, which were comparable with previous studies. The reported incidence of PH ranged from 7 to 16.8% in patients with reperfusion therapy (9, 10, 21, 22). The incidence of SICH after IVT ranged from 2 to 7% and that after endovascular thrombectomy was about 4.4% in clinical trials and stroke registries (3, 23, 24).

This study demonstrated that the admission FDP was associated with PH, even after adjusting the baseline NIHSS score and endovascular thrombectomy. Trouillas et al. and Sun et al. failed to find a positive association between the admission FDP level and PH (9, 10). We thought that it was due to the small sample sizes that only 11 and 6 patients had PH in their studies, respectively. The FDP, as the fragments of fibrin and fibrinogen, includes powerful anticoagulants. Moreover, the FDP can impair platelet function by interfering with platelet ATP release, aggregation, and signaling (25). For these reasons, patients with high levels of the FDP at admission were more likely to experience PH after IVT.

A previous study also found that a higher level of the FDP was associated with unfavorable stroke outcome (26). Nevertheless, patients in this study were not restricted to those who received IVT and the blood sample was collected within 72 h after stroke (26). Zhu et al. found that the admission FDP levels correlated with the NIHSS score 1 h after IVT (11). However, they excluded patients who have large vessel occlusion and underwent endovascular thrombectomy. This study found that the admission FDP > 3.085 μg/ml could predict unfavorable outcome after adjusting the baseline NIHSS score, endovascular thrombectomy, and PH. However, we failed to demonstrate that this relationship was mediated by PH. So, other mechanisms must exist. It was found that the FDP was involved in leukocyte transendothelial migration through the very low-density lipoprotein (VLDL) receptor-dependent pathway (27). Leukocytes could damage the endothelial, blood–brain barrier and promote inflammation response and reperfusion injury (28). The above mechanisms might account for our results that the FDP was positively associated with unfavorable outcome after IVT in patients with AIS.

With the extension of the therapeutic time window, reperfusion therapy is applicable to more and more patients. The FDP assay before reperfusion therapy should be a standard practice to detect the patients at risk for PH and who should be monitored with more attention. Also, study should be done to answer the reason why the elevated FDP is associated with unfavorable stroke outcomes.

This study had several limitations. First, the single-center, hospital-based retrospective design might have a potential risk of selection bias. Second, the sample size was insufficient and might influence the statistical power of the results. Studies based on a larger population were needed.

In summary, this study demonstrated that the admission FDP level might be used as a predictor of PH and unfavorable outcome in patients with AIS after IVT. Further prospective studies are needed to confirm this finding.

## Data Availability Statement

The raw data supporting the conclusions of this article will be made available by the authors, without undue reservation.

## Ethics Statement

The studies involving human participants were reviewed and approved by the Human Ethics Committee of the Second Affiliated Hospital of Chongqing Medical University. The patients/participants provided their written informed consent to participate in this study.

## Author Contributions

CL drafted and revised the manuscript, participated in study concept and design, conducted the statistical analyses, and interpreted the data. YZ and LN participated in data collection and interpretation. JL participated in the data analysis. All authors contributed to the article and approved the submitted version.

## Funding

This work was supported by the Medical Scientific Research Project of Chongqing Municipal Health commission (Grant No. 2019MSXMO17).

## Conflict of Interest

The authors declare that the research was conducted in the absence of any commercial or financial relationships that could be construed as a potential conflict of interest.

## Publisher's Note

All claims expressed in this article are solely those of the authors and do not necessarily represent those of their affiliated organizations, or those of the publisher, the editors and the reviewers. Any product that may be evaluated in this article, or claim that may be made by its manufacturer, is not guaranteed or endorsed by the publisher.
