# Supplementary material for: High Level of the Fibrin Degradation Products at Admission Predicts Parenchymal Hematoma and Unfavorable Outcome of Ischemic Stroke After Intravenous Thrombolysis
Source: Front Neurol. 2022 Jan 18;12:797394. doi: 10.3389/fneur.2021.797394 (PMC8803633; doi:10.3389/fneur.2021.797394)
Supplement: Supplementary file 1 [file Data_Sheet_1.docx]

Supplementary Material

**Supplemental Table 1** Multivariable logistic regression analysis for parenchymal hematomas

|  | OR | 95%CI | *P* value |
| --- | --- | --- | --- |
| Hypertension | 0.488 | 0.195-1.224 | 0.126 |
| Baseline NIHSS score | 1.063 | 0.989-1.143 | 0.098 |
| FDP, μg/ml | 1.034 | 1.000-1.069 | 0.047 |
| Endovascular thrombectomy | 4.553 | 1.711-12.115 | 0.002 |

NIHSS: national institute of health stroke scale; FDP: fibrin degradation products.

**Supplemental Table 2** Multivariable logistic regression analysis for unfavorable outcome

|  | OR | 95%CI | *P* value |
| --- | --- | --- | --- |
| Model 1 | | | |
| Age, y | 1.015 | 0.980-1.051 | 0.406 |
| Male | 0.744 | 0.339-1.633 | 0.461 |
| Hypertension | 0.661 | 0.297-1.470 | 0.310 |
| Atrial fibrillation | 0.871 | 0.359-2.118 | 0.761 |
| Baseline NIHSS score | 1.177 | 1.089-1.273 | ＜0.001 |
| FDP, μg/ml | 1.026 | 0.968-1.087 | 0.391 |
| INR | 0.394 | 0.055-2.849 | 0.356 |
| PT, s | 1.029 | 0.936-1.131 | 0.554 |
| Endovascular thrombectomy | 1.569 | 0.565-4.358 | 0.388 |
| PH | 4.729 | 1.433-15.601 | 0.011 |
| Model 2 | | | |
| Age, y | 0.988 | 0.950-1.027 | 0.531 |
| Male | 0.765 | 0.332-1.765 | 0.531 |
| Hypertension | 0.677 | 0.287-1.596 | 0.372 |
| Atrial fibrillation | 0.705 | 0.278-1.787 | 0.461 |
| Baseline NIHSS score | 1.185 | 1.091-1.288 | ＜0.001 |
| INR | 0.225 | 0.025-2.049 | 0.186 |
| PT, s | 1.053 | 0.955-1.162 | 0.299 |
| Endovascular thrombectomy | 1.624 | 0.525-5.017 | 0.400 |
| PH | 3.639 | 1.065-12.441 | 0.039 |
| FDP＞3.085μg/ml | 7.086 | 2.818-17.822 | ＜0.001 |

NIHSS: national institute of health stroke scale; FDP: fibrin degradation products; INR: international normalized ratio; PT: prothrombin time; PH: parenchymal hematomas.

**Supplemental Table 3** Univariate comparison between hemorrhagic transformation (HT) group and non-HT group

|  | non-HT(n=138) | HT(n=43) | *P* value |
| --- | --- | --- | --- |
| Age, y | 72.0(64.0-79.0) | 74.0(61.0-81.0) | 0.956 |
| Male, n(%) | 80(58.0) | 22(51.2) | 0.432 |
| Hypertension, n(%) | 102(73.9) | 23(53.5) | 0.011 |
| Diabetes, n(%) | 42(30.4) | 12(27.9) | 0.752 |
| Hyperlipemia, n(%) | 93(67.4) | 24(55.8) | 0.166 |
| Atrial fibrillation, n(%) | 48(34.8) | 25(58.1) | 0.006 |
| Smoking, n(%) | 57(41.3) | 15(34.9) | 0.453 |
| Baseline NIHSS score | 7.0(4.0-13.0) | 13(7.0-19.0) | ＜0.001 |
| FDP, μg/ml | 2.2(1.5-3.3) | 3.2(1.8-5.4) | 0.014 |
| International normalized ratio | 0.98(0.93-1.04) | 1.03(0.98-1.09) | 0.006 |
| Prothrombin time, s | 13.0(12.5-13.7) | 13.5(12.8-14.2) | 0.009 |
| Activated partial thromboplastin time, s | 34.4(32.2-36.8) | 35.0(32.0-38.6) | 0.338 |
| Endovascular thrombectomy, n(%) | 15(10.9) | 20(46.5) | ＜0.001 |

NIHSS: national institute of health stroke scale; FDP: fibrin degradation products.

**Supplemental Table 4** Multivariable logistic regression analysis for hemorrhagic transformation

|  | OR | 95%CI | *P* value |
| --- | --- | --- | --- |
| Hypertension | 0.541 | 0.241-1.214 | 0.136 |
| Atrial fibrillation | 1.910 | 0.814-4.485 | 0.137 |
| Baseline NIHSS score | 1.052 | 0.983-1.125 | 0.143 |
| FDP, μg/ml | 1.027 | 0.995-1.059 | 0.098 |
| Endovascular thrombectomy | 5.676 | 2.348-13.722 | ＜0.001 |

NIHSS: national institute of health stroke scale; FDP: fibrin degradation products.
